# Interdependence of the key molar ratios (SiO_2_/Al_2_O_3_ and Al_2_O_3_/Na_2_O) in metakaolin-based geopolymers: phase composition, microstructure and mechanical insights

**DOI:** 10.1039/d6ra04380a

**Published:** 2026-07-17

**Authors:** Hisham Abdeen, Alaa Mohsen, AbdelMonem Soltan, Mohamed Kohail

**Affiliations:** a Faculty of Engineering, Ain Shams University Cairo Egypt alaa.mohsen@eng.asu.edu.eg; b University College of Science and Technology Palestine; c Geology Department, Faculty of Science, Ain Shams University Cairo 11566 Egypt

## Abstract

Recently, metakaolin-geopolymers have gained increasing importance as alternative building materials to ordinary Portland cement. Intensive research published in this field has revealed several contradictions concerning the oxide's molar ratios (SiO_2_/Al_2_O_3_ and Al_2_O_3_/Na_2_O) that influence the properties of the metakaolin geopolymers, complicating the understanding of the geopolymerization process. Accordingly, the main objective of this study is to resolve this discrepancy by measuring the impact of a wide range of SiO_2_/Al_2_O_3_ (2.74 to 4.1) and Al_2_O_3_/Na_2_O (0.75 to 1.50) molar ratios on the mechanical performance of the metakaolin geopolymers. In all specimens, H_2_O/Na_2_O was fixed at 9.50, and they were cured for 24 h at 60 °C and then kept in a humidity of 20 °C ± 5 °C for 28 days. The results were confirmed by XRD, FTIR spectroscopy, TGA/DTG, SEM/EDX and texture characteristics analyses. The data revealed that the mix-design, using SiO_2_/Al_2_O_3_, Al_2_O_3_/Na_2_O and water/solid ratios of 3.5, 1.40 and 0.32, respectively, achieves a superior mechanical performance (50.80 MPa). This mix-design showed that it was composed of thermally stable three-dimensional geopolymeric phases and the lowest amount of unreacted metakaolin. Its microstructure and texture characteristics reveal dense, interconnected and well-dispersed strength-giving-phases. Finally, it can be concluded that the quality of the geopolymer depends on the SiO_2_/Al_2_O_3_ ratio, followed by the Al_2_O_3_/Na_2_O ratio, as SiO_2_/Al_2_O_3_ controls the nature of the formed geopolymeric phase. The main roles of Na_2_O in the geopolymeric matrix are the charge balancing of [Al(OH)_4_]^−^ and contributing to the alkalinity required for metakaolin dissolution. Therefore, the claim made in some previous studies that Al_2_O_3_/Na_2_O = 1 is universally optimal is inaccurate as the value of this ratio must depend on the SiO_2_/Al_2_O_3_ ratio.

## Abstract 2

Recently, metakaolin-geopolymers have gained increasing importance as alternative building materials to ordinary Portland cement.

## Graphical abstract

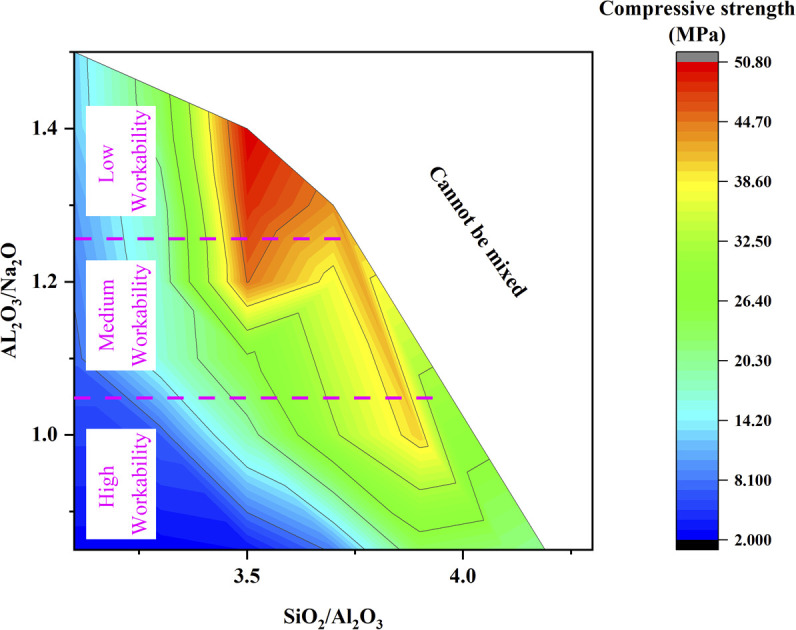

## 1. Introduction

Geopolymer concrete has received considerable attention in the construction industry because of the benefits of utilizing natural clays or industrial waste as well as reducing greenhouse gas emissions.^1–3^ It possesses excellent mechanical properties and superior durability, including fire- and acid-resistance.^4^ Geopolymers are synthesized by the alkali activation of aluminosilicates using an alkaline activator at room temperature or higher.^5–7^ Geopolymerization involves the dissolution of aluminosilicate oxides in alkali solutions, polycondensation and polymerization, forming the Si–O–Al–O bonds.^8–10^

Metakaolin is an aluminosilicate-rich precursor that is widely used in geopolymer production owing to its high reactivity.^11,12^ It is produced by calcining natural kaolin between 550 °C and 750 °C.^13,14^ Heating to higher temperatures can lead to recrystallization and the formation of mullite or spinel, resulting in the loss of pozzolanicity.^15–17^ Sodium hydroxide (NaOH) is most commonly used to prepare alkali activator solutions for geopolymer synthesis due to its low production cost.^18,19^ The combination of NaOH and sodium silicate (Na_2_SiO_3_) provides more interesting properties than those exhibited by using the alkali source alone.^20,21^

Most published studies on the metakaolin geopolymers have focused on the following parameters that affect the compressive strength properties: kaolin calcination conditions;^22,23^ curing conditions;^24–27^ alkaline-activator type, concentration, and ratio;^28–30^ mass ratio of the alkali activator solution to source material.^31^ These parameters are translated into variations in the SiO_2_/Al_2_O_3_ molar ratio,^32^ Al_2_O_3_/Na_2_O molar ratio,^33^ SiO_2_/Na_2_O molar ratio,^34,35^ H_2_O/Na_2_O molar ratio,^36^ and water/solids ratio.^37^

It was found that the compressive strength and microstructural evolution of metakaolin geopolymers strongly depend on their SiO_2_/Al_2_O_3_ and Al_2_O_3_/Na_2_O molar ratios.^33,38,39^ Different researchers have reported different views of the SiO_2_/Al_2_O_3_ and Al_2_O_3_/Na_2_O ratios.^34,40–43^ Ghanbari *et al.*^34^ reported that the optimum molar ratios of SiO_2_/Al_2_O_3_ and Al_2_O_3_/Na_2_O reached 2.9 and 1.72, respectively. This is different from the results obtained by Silva *et al.*,^40^ which suggested using SiO_2_/Al_2_O_3_ and Al_2_O_3_/Na_2_O in the range of 3.4–3.8 and 0.8–1.0, respectively. Yunsheng *et al.*^43^ showed that the highest compressive strength of 34.9 MPa was obtained for SiO_2_/Al_2_O_3_ = 5.5 and Al_2_O_3_/Na_2_O = 1.0. Table 1 summarizes previous studies that used different mixing molar ratios to achieve the highest compressive strength values of the metakaolin geopolymers. The optimum calcination conditions to transform kaolin into metakaolin and curing regimes are also illustrated.

**Table 1:** Previous literature on metakaolin geopolymer fabrication

Reference	Molar ratios			Water/solids	Calcination conditions	Curing conditions	Compressive strength (MPa)
	SiO_2_/Al_2_O_3_	Al_2_O_3_/Na_2_O	H_2_O/Na_2_O				
Ghanbari *et al.*^34^	2.9	1.72	13.75	0.45	750 °C for 24 h	85 °C for 24 h	65.9 (28 days)
Lizcano *et al.*^41^	3	1	11	0.56	—	80 °C for 24 h	32.1 (1 day)
Tchakouté *et al.*^44^	3	—	10	—	700 °C for 4 h	60 °C for 24 h	63.8 (28 days)
Kong *et al.*^45^	3.08	1.52	—	—	750 °C for 6 h	80 °C for 24 h	45 (3 days)
Riahi *et al.*^46^	3–3.5	0.9–1	10–12.5	0.61	—	50 °C for 24 h	60.6 (7 days)
Mo *et al.*^47^	3.3	1.11	10.56	0.47	800 °C for 3 h	60 °C for 7 days	97.95 (7 days)
Barbosa *et al.*^48^	3.3	1.2	10	0.41	700 °C for 6 h	65 °C for 2.5 h	49 (3 days)
Silva *et al.*^40^	3.4–3.8	0.8–1.0	13.6	0.61–0.65	—	40 °C for 72 h	22 (3 days)
Kamalloo *et al.*^49^	3.6–3.8	0.83–1	10–11	0.49	750 °C for 4 h	70 °C for 4 h	80 (7 days)
Duxson *et al.*^39^	3.8	1	11	0.49	—	40 °C for 24 h	82 (7 days)
Lahotia *et al.*^33^	4	0.91	11	0.52	—	25 °C for 24 h	72.51 (7 days)
Ozer *et al.*^32^	4.4	—	—	—	700 °C for 1 h	60 °C for 24 h	23 (28 days)
Rowles and O'Connor^38^	5	0.78	11.64	0.55	750 °C for 24 h	75 °C for 24 h	64 (7 days)
Yunsheng *et al.*^43^	5.5	1	7	0.26	700 °C for 12 h	20 °C for 28 days	34.9 (28 days)

According to the previous literature, there are conflicting results on the molar ratios of SiO_2_/Al_2_O_3_ and Al_2_O_3_/Na_2_O that are required to achieve the highest mechanical performance. Therefore, this study aims to resolve this controversy by examining the effect of a wide range of ratios on the compressive strength values. The results are then verified using analysis techniques, including phase identification (XRD and TGA/DTG) and microstructure texture analysis (Brunauer–Emmett–Teller model (BET), Barrett–Joyner–Halenda model (BJH), and SEM), to confirm the optimal ratios.

## 2. Materials and methods

### 2.1. Materials

The kaolin sample used in this work was Egyptian kaolin clay collected from the Sinai region. Metakaolin was prepared by crushing raw kaolin clay using a jaw crusher and then milling it in a ball mill. The calcination-temperature range for preparing metakaolin was determined using thermogravimetric analysis (TGA/DTG). The optimal temperature was identified by calculating the dehydroxylation degree (*D*, %), an essential indicator of kaolin's conversion into metakaolin during thermal treatment. Based on the values of mass-loss% at specific temperature (*M*) and total mass-loss% resulting from the complete dehydroxylation process (*M*_max_), measured from TGA, the *D* (%) was calculated, as in eqn (1). The *M*_max_ is measured from TGA when the mass-loss becomes nearly constant.^50,51^ TGA/DTG analysis was performed using a NETZSCH/STA-449F5 instrument; 20 mg of the sample was filled in a platinum crucible, then heated from 30 to 1000 °C with an operating heating rate of 20 °C min^−1^ in N_2_ gas medium.1
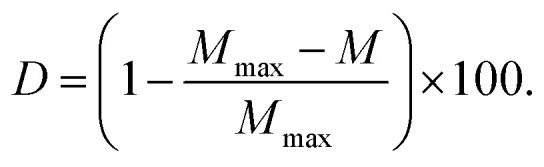


The expected calcination temperature was confirmed by examining the phase composition and microstructure of kaolin before and after calcination using X-ray diffraction (XRD) and scanning electron microscopy (SEM), respectively. XRD analysis was carried out using a Philips/Xpert-2000 with 40 kV power, an X-ray with *λ* = 1.54 Å, Cu Kα radiation, a scanning range of 2*θ* = 2°–60°, and a scanning speed of 0.02° sec^−1^. SEM imaging was conducted using a Thermo-Scientific/Quattro-S with a voltage of 20 kV. Furthermore, X-ray fluorescence (XRF) was employed to study the impact of calcination temperature on the mineralogical-oxide wt% of kaolin. XRF was obtained using a Xios, stylePW-1400, in which 10 g of the sample was mixed with 1 g of borax (filling material) and compressed in a lead ring at 3–12 tons per cm^3^.

Sodium hydroxide (NaOH) and sodium silicate (Na_2_SiO_3_) solutions were used to prepare alkaline activator solutions. NaOH pellets with a purity of 98% and a Na_2_SiO_3_ solution with the chemical composition 30.12 wt% SiO_2_, 11.42 wt% Na_2_O, and 58.46 wt% H_2_O (modulus ratio: SiO_2_/Na_2_O = 2.6) were purchased from El-Gomhouria Chemical Company.

### 2.2. Specimen preparation and testing

Generally, the metakaolin geopolymer with superior characteristics is obtained by achieving optimum mixing molar ratios of the casting specimens (SiO_2_/Al_2_O_3_ and Al_2_O_3_/Na_2_O). Accordingly, thirty-two metakaolin-based geopolymeric specimens were prepared using different concentrations of NaOH solutions and various Na_2_SiO_3_/NaOH ratios. The design included nine main groups, depending on the SiO_2_/Al_2_O_3_ ratio, which ranged from 2.74 to 4.1. The group with the same SiO_2_/Al_2_O_3_ ratio contains different Al_2_O_3_/Na_2_O ratios, ranging from 0.75 to 1.50. Based on the preliminary study, specimens with very low workability (at high Al_2_O_3_/Na_2_O) were not cast; also, specimens with very low compressive strength values (at low Al_2_O_3_/Na_2_O) were ignored. The molar ratio of H_2_O/Na_2_O for all mixtures in this research was constant at 9.50. Furthermore, two supplementary mixes (mixes #33 and 34) in which H_2_O/Na_2_O ratios of 12.0 were prepared for use in arranging the factors affecting the compressive strength (Al_2_O_3_/Na_2_O and water/solid ratios). After preparing and cooling the alkaline activator solutions, they are mixed with the metakaolin powder in a Hobart mixer for 5 min. The workability of the obtained fresh geopolymeric pastes was evaluated. Subsequently, they were cast into 1-inch cubic molds, kept at 20 °C ± 5 °C and relative humidity of 95% ± 5% until demolding. The hardened specimens were then cured at the same humidity in an oven at 60 °C for 24 h. The curing process was continued at 20 °C ± 5 °C and 95% ± 5% relative humidity again for 28 days.

The average compressive strength of three replicate cubes from each specimen was measured using a compression testing machine (control, maximum load of 250 kN) according to ASTM C109M-20.^52^ After that, the fractured specimens were ground for analysis using XRD, Fourier-transform infrared spectroscopy (FTIR) and TGA/DTG to identify the phase composition of the formed strength-giving-phases. FTIR analysis was performed using a Mattson-1000 Unicam instrument in the range of 400–4000 cm^−1^. The texture parameters were analyzed using a pore-size distribution and surface-area analyzer (MICROTRAC-BELSORP/MINI-X). The surface area (SA) was calculated using the Brunauer–Emmett–Teller (BET) model. The pore size characteristics (total pore volume (*V*_t_), adsorption capacity (*V*_m_), and maximum pore diameter (dp_max_)) were calculated using the Barrett–Joyner–Halenda (BJH) model. For this analysis, a 0.5 g sample was placed in a quartz cell under 10^−6^ Pa vacuum at 80 °C for 4 h to outgas; the cell was then transferred to the equipment for N_2_ adsorption–desorption measurements at 77 K. The microstructure of the geopolymeric matrix was examined directly on the fractured specimen using a SEM equipped with an energy-dispersive X-ray (EDX) instrument.

## 3. Results and discussion

### 3.1. Calcination of kaolin to metakaolin

**Fig. 1:**
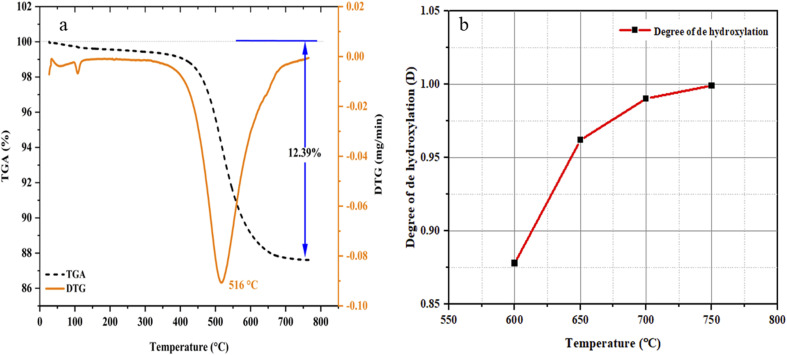
(a) TGA/DTG curves and (b) dehydroxylation degree of kaolin.

To identify the optimum calcination temperature of the kaolin sample to transform into highly reactive metakaolin, its thermal behavior was examined, as represented in Fig. 1a. The TGA/DTG curve shows that a significant mass loss appeared at 400–700 °C, accompanied by an endothermic peak centered at 516 °C, which is associated with dehydroxylation of kaolin, as also noted by Aziz *et al.*^53^ Regarding the measurement of the dehydroxylation degree (*D*, %), it can be observed that there is no significant change in the mass-loss% in the TGA starting from 700 °C, indicating that *M*_max_ is approximately 12.39%. Fig. 1b shows that, upon increasing the temperature from 600 to 700 °C, the *D*% increases from 87.88% to 99.03%. These data show that near-complete dehydroxylation of the kaolin sample can be achieved at 700 °C for 1 h, as the *D* reaches 99.03%, exceeding the value of 95% that is required to produce highly reactive cementitious materials for concrete applications.^45^ Considering temperatures beyond 700 °C, although *D*% increased to 99.92% at 750 °C, this was not suitable from an economic point of view.^51^

**Fig. 2:**
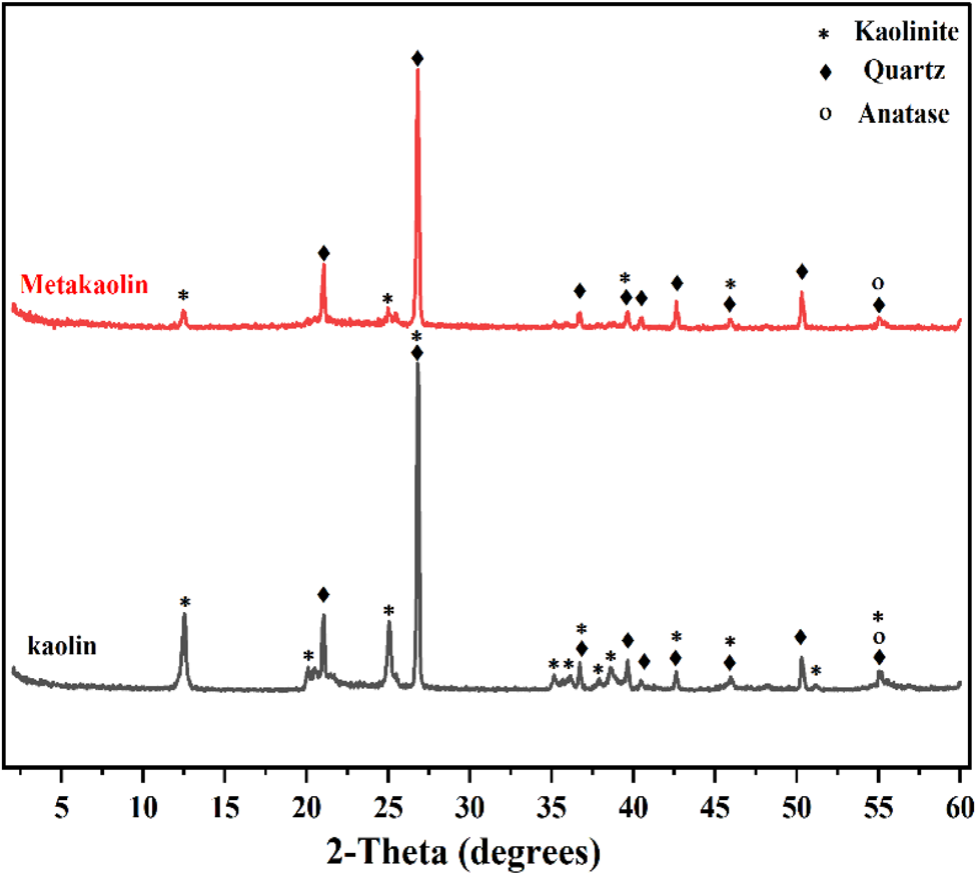
XRD patterns of kaolin and metakaolin.

XRD patterns of the kaolin and metakaolin obtained by thermal treatment of kaolin at 700 °C for 1 h were analyzed to confirm that this calcination condition is sufficient to transform the kaolin into metakaolin. Fig. 2 shows that the major crystalline phases of the kaolin sample are kaolinite (Al_2_Si_2_O_5_(OH)_4_, PDF# 00-012-0447, 2*θ* = 12.5°, 20.0°, 25.1°, 35.2°, 36.2°, 37.8°, 38.6°, 39.6°, 42.6°, 51.2° and 55.1°), quartz (SiO_2_, PDF# 01-074-3485, 2*θ* = 21.0°, 26.8°, 36.7°, 39.6°, 40.5°, 42.6°, 46.0°, 50.3° and 55.1°) and anatase (TiO_2_, PDF# 01-071-1167, 2*θ* = 55.1°). After calcination, a significant reduction in the intensities of some peaks related to the kaolinite phase, as well as the disappearance of others, such as at 2*θ* = 20.0°, 35.2°, 36.2°, 37.8°, 38.6°, 42.6°, 51.2° and 55.1°, shows that kaolin has been converted into metakaolin.^22,54,55^

**Fig. 3:**
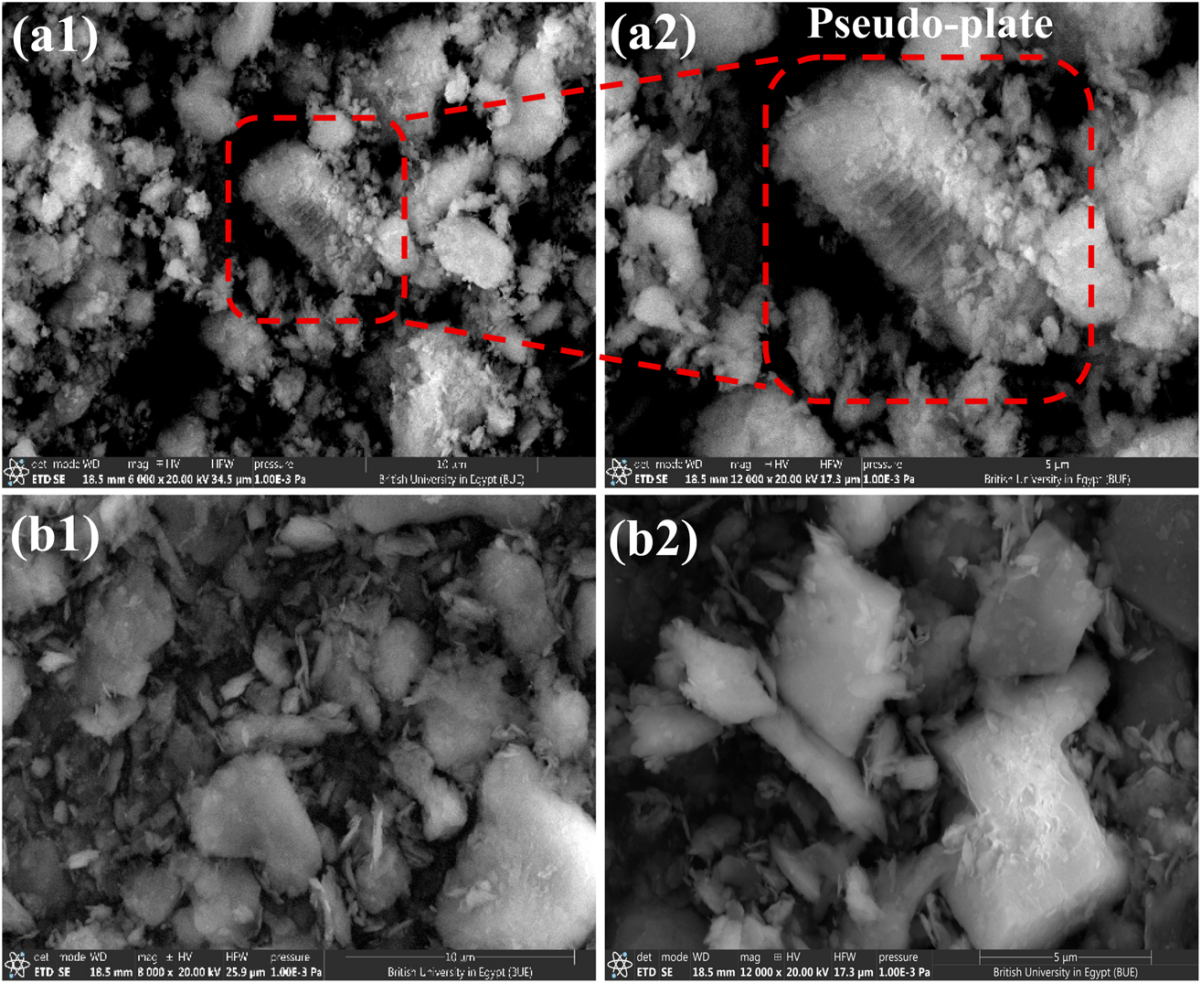
SEM images of (a1 and a2) kaolin and (b1 and b2) metakaolin.

The effect of calcination temperature on the morphology of kaolin's phases was also examined, as shown in Fig. 3a and b. The SEM image of kaolin (Fig. 3a1 and 2) revealed the presence of pseudo-plate clusters, which are characteristic of kaolinite. Conversely, the SEM image of metakaolin (Fig. 3b1 and 2) displays a separation between the plates, illustrating the role of calcination in the dehydroxylation of kaolinite.^14,55^ As this study aimed to investigate the impact of SiO_2_/Al_2_O_3_ and Al_2_O_3_/Na_2_O on the performance of the prepared geopolymers, the SiO_2_, Al_2_O_3_, and Na_2_O wt% in metakaolin were measured using XRF, as shown in Table 2.

**Table 2:** Chemical composition of kaolin and metakaolin

Oxide	SiO_2_	TiO_2_	Al_2_O_3_	Fe_2_O_3_	MnO	MgO	CaO	Na_2_O	K_2_O	P_2_O_5_	Cl	SO_3_
Kaolin	62.27	1.87	26.91	0.98	<0.01	0.07	0.09	<0.01	<0.01	<0.01	<0.01	<0.01
Metakaolin	56.18	2.63	34.76	1.46	0.02	0.21	0.97	<0.01	<0.01	<0.01	<0.01	0.11

The same procedure was used before by Souri *et al.*^22^ to measure the optimum calcination temperature of Iranian kaolin powder by investigating the pozzolanic activity. They calcined kaolin at different temperatures from 600 °C to 850 °C for 3 h and concluded that calcination at 700 °C is the optimum firing temperature (the highest amorphousity); metakaolin recrystallized at higher temperatures.^14^ Another study used the measured heat evolved during the polycondensation process to measure the optimum firing conditions. Cioffi *et al.*^56^ found that the heat evolved is low in the case of kaolin fired at 550 °C for 2 h, while it was very high in the case of firing at 650 °C for the same period. From these results, they predicted that firing kaolin at 650 °C for 2 h is the optimum condition among firing at 500 °C, 550 °C, 650 °C and 700 °C for 2, 4 and 6 h.^56^ From the above discussion, it is concluded that the calcination process at 700 °C for 1 h transformed the crystalline kaolin into a more reactive aluminosilicate precursor that is better suited for geopolymerization.

### 3.2. Workability

**Fig. 4:**
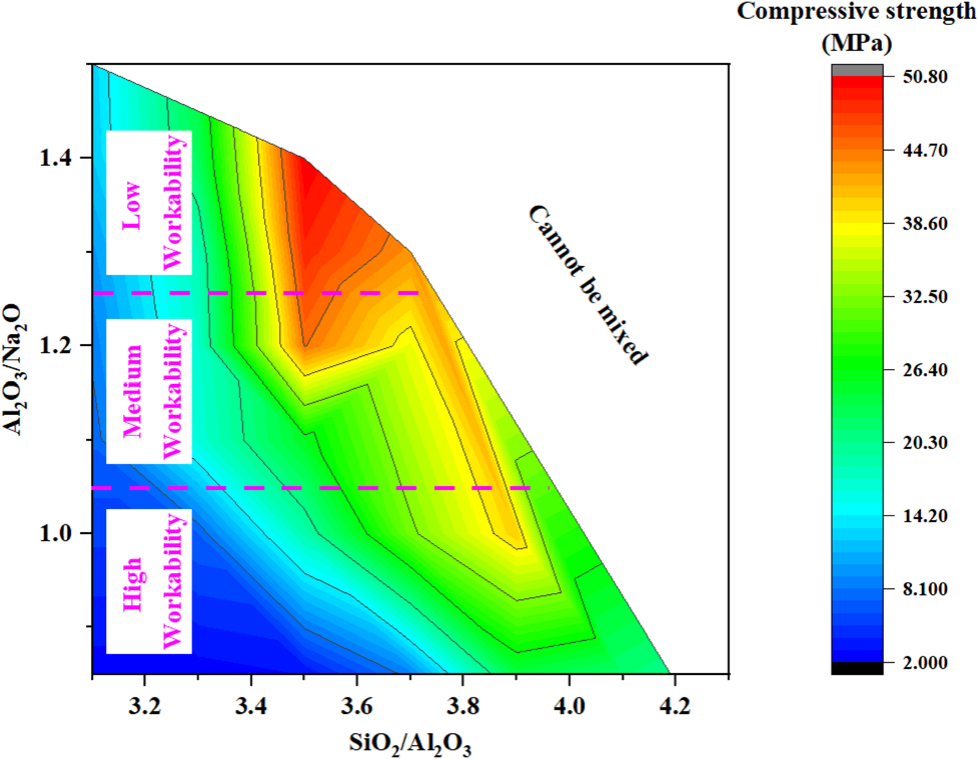
Synergistic effects of the SiO_2_/Al_2_O_3_ and Al_2_O_3_/Na_2_O molar ratios on the 28-day compressive strength (MPa) of metakaolin geopolymers at H_2_O/Na_2_O = 9.50.

Generally, the fresh behavior of metakaolin-based geopolymers depends on the workability, which directly influences the feasibility of casting, compaction, and finishing.^57–59^ In alkali-activated systems, these properties are influenced by the alkaline activator type/concentration, which affects the kinetics of the geopolymerization process. Also, increasing the water/solids ratio usually enhances the initial workability of the alkali-activated binders because it reduces solids packing and interparticle friction.^60–63^Fig. 4 illustrates the clear variation in observed workability during the casting process, ranging from high for Al_2_O_3_/Na_2_O ratios of 1.0 or lower, to medium for ratios between 1.1 and 1.2, and finally too low for ratios ranging from 1.3 to 1.5. This trend corresponds with a systematic decrease in the water/solid ratio as the molar ratio of Al_2_O_3_/Na_2_O increases, as shown in Table 3.^60,64,65^ To adjust the Al_2_O_3_/Na_2_O ratio across different mixes while maintaining a specific SiO_2_/Al_2_O_3_ ratio, it is necessary to modify the amount of Na_2_O used in mixes #1–32. Additionally, the quantity of water used to prepare the alkaline solution must be adjusted simultaneously to keep the H_2_O/Na_2_O ratio at 9.5 for all mixes. As a result, the water/solid ratio varied. Preparation processes for mixes where the water/solid ratio is less than 0.32 have proven unsuccessful. This is primarily due to a significant decline in workability when the Al_2_O_3_/Na_2_O ratio exceeds 1.5, 1.45, 1.4 and 1.3 for groups 3, 4, 5, and 6, respectively. Additionally, mixes with high SiO_2_/Al_2_O_3_ and Al_2_O_3_/Na_2_O ratios, such as combinations of 4.3 and 1.0 or 4.1 and 1.0, respectively, were not prepared, even though their water/solid ratio was above 0.32. This was because it is challenging to create NaOH solutions with concentrations greater than 16 M, which are necessary for these samples. Further increases in NaOH concentrations make the solution more difficult to prepare and handle due to its increased viscosity and the higher probability of sodium hydroxide crystallization at room temperature, resulting in degraded compressive strength.^66,67^ All the mixture designs are listed in Table 3 with sodium hydroxide (NaOH) concentrations ranging from 7.96 to 14.18 M.

**Table 3:** Mix-design of the prepared metakaolin geopolymers and their compressive strength after 28 days

Mix no.	MK mass (g)	NaOH solution mass (g)	Na_2_SiO_3_ solution mass (g)	# M NaOH (mol)	Liquid/solid	Na_2_SiO_3_/NaOH	SiO_2_/Al_2_O_3_	Al_2_O_3_/Na_2_O	H_2_O/Na_2_O	Water/solids	Compressive strength (MPa)
1	300	233.7	0.00	7.96	0.78	0.00	2.74	1.10	9.50	0.44	2.08 ± 0.16
2	300	214.2	0.00	7.96	0.71	0.00	2.74	1.20	9.50	0.41	4.88 ± 0.08
3	300	197.7	0.00	7.96	0.66	0.00	2.74	1.30	9.50	0.39	6.56 ± 0.16
4	300	210.0	32.0	8.29	0.81	0.15	2.90	1.10	9.50	0.43	4.00 ± 0.16
5	300	190.6	32.0	8.32	0.74	0.17	2.90	1.20	9.50	0.40	5.65 ± 0.16
6	300	174.1	32.0	8.36	0.69	0.18	2.90	1.30	9.50	0.38	6.75 ± 1.79
7	300	180.3	72.8	8.82	0.84	0.40	3.10	1.10	9.50	0.42	7.28 ± 0.40
8	300	161.1	72.8	8.92	0.78	0.45	3.10	1.20	9.50	0.39	8.48 ± 0.48
9	300	144.3	72.8	9.04	0.72	0.51	3.10	1.30	9.50	0.36	9.84 ± 1.04
10	300	130.2	72.8	9.16	0.68	0.56	3.10	1.40	9.50	0.34	12.16 ± 1.33
11	300	118.0	72.8	9.28	0.64	0.62	3.10	1.50	9.50	0.32	12.56 ± 1.04
12	300	219.1	113.7	9.07	1.11	0.52	3.30	0.85	9.50	0.50	2.40 ± 0.48
13	300	173.7	113.7	9.37	0.96	0.66	3.30	1.00	9.50	0.44	7.09 ± 1.09
14	300	150.3	113.7	9.59	0.88	0.76	3.30	1.10	9.50	0.41	16.53 ± 1.39
15	300	130.8	113.7	9.83	0.82	0.87	3.30	1.20	9.50	0.38	17.20 ± 4.56
16	300	114.3	113.7	10.10	0.76	1.00	3.30	1.30	9.50	0.35	18.19 ± 2.77
17	300	99.9	113.7	10.43	0.71	1.14	3.30	1.40	9.50	0.33	22.52 ± 1.65
18	300	94.00	113.7	10.56	0.69	1.21	3.30	1.45	9.50	0.32	23.52 ± 2.40
19	300	188.9	154.5	9.72	1.15	0.82	3.50	0.85	9.50	0.49	3.04 ± 0.16
20	300	144.4	154.5	10.26	1.00	1.07	3.50	1.00	9.50	0.43	18.56 ± 1.28
21	300	120.7	154.5	10.70	0.92	1.28	3.50	1.10	9.50	0.39	25.33 ± 2.67
22	300	100.4	154.5	11.27	0.85	1.54	3.50	1.20	9.50	0.37	44.85 ± 1.71
23	300	84.6	154.5	11.86	0.80	1.83	3.50	1.30	9.50	0.34	48.48 ± 0.96
24	300	70.3	154.5	12.68	0.75	2.20	3.50	1.40	9.50	0.32	50.80 ± 2.30
25	300	158.7	195.4	10.63	1.18	1.23	3.70	0.85	9.50	0.47	8.64 ± 0.32
26	300	113.8	195.4	11.65	1.03	1.72	3.70	1.00	9.50	0.41	31.92 ± 4.40
27	300	71.0	195.4	13.86	0.89	2.75	3.70	1.20	9.50	0.35	37.36 ± 0.24
28	300	55.0	195.4	15.53	0.83	3.55	3.70	1.30	9.50	0.33	43.20 ± 3.10
29	300	129.8	236.1	11.84	1.22	1.82	3.90	0.85	9.50	0.46	23.79 ± 1.65
30	300	84.3	236.1	13.95	1.07	2.80	3.90	1.00	9.50	0.40	40.44 ± 0.40
31	300	99.6	276.9	13.92	1.26	2.78	4.10	0.85	9.50	0.45	21.49 ± 2.59
32	300	109.9	317.6	14.18	1.43	2.89	4.30	0.75	9.50	0.49	15.31 ± 1.09
33	300	120.2	154.5	8.36	0.92	1.28	3.50	1.30	12.0	0.43	41.57 ± 2.78
34	300	89.1	195.4	9.50	0.95	2.20	3.70	1.30	12.0	0.41	40.84 ± 0.16

### 3.3. Mechanical performance

#### 3.3.1. Compressive strength

To obtain a comprehensive view concerning the effect of molar ratios (SiO_2_/Al_2_O_3_ and Al_2_O_3_/Na_2_O) on the mechanical properties of metakaolin-based geopolymers, thirty-two mixes were used to assess these factors. The details of the molar ratios for all parameters are listed in Table 3. The H_2_O/Na_2_O ratio determines the alkalinity of the system, which affects the degree of geopolymerization.^46^ Therefore, the H_2_O/Na_2_O molar ratio was considered constant at 9.5 for all specimens. Fig. 4 shows a contour plot that describes the synergistic impact of SiO_2_/Al_2_O_3_ and Al_2_O_3_/Na_2_O molar ratios on the compressive strength at 28 days. The compressive strength contour plot was generated from the experimental *XYZ* dataset, where the SiO_2_/Al_2_O_3_ molar ratio, Al_2_O_3_/Na_2_O molar ratio and compressive strength were used as the *X*, *Y* and *Z* variables, respectively. Because the experimental setup did not create a completely uniform rectangular grid, the contour surface was generated through triangulation-based interpolation of the measured compressive strength data. The interpolation was applied within the workable composition region only. While compositions that could not be mixed, as mentioned above, were considered as missing/non-valid points.

The general trend shows that the mixes in the main group with a high SiO_2_/Al_2_O_3_ ratio have the highest compressive strength compared to the other groups, reaching a peak at about a 3.5 ratio. A greater rise in the SiO_2_/Al_2_O_3_ ratio results in decreasing compressive strength again. The highest strength values achieved at SiO_2_/Al_2_O_3_ ratios of 2.74, 2.90, 3.10, 3.30, 3.50, 3.70, 3.90, 4.10 and 4.30 are 6.56, 6.75, 12.56, 23.52, 50.80, 43.20, 40.44, 21.49 and 15.31 MPa, respectively. The low strength of specimens with low SiO_2_/Al_2_O_3_ molar ratios is probably due to the consequent high Al_2_O_3_, resulting in the formation of less connected geopolymeric gel products, with a weak and brittle structure of low hydraulic nature.^40^ A low SiO_2_/Al_2_O_3_ ratio results in a less cross-linked, two-dimensional network rather than the more robust three-dimensional network that offers strength and durability. Conversely, as the SiO_2_/Al_2_O_3_ ratio increases, three-dimensional network structures become dominant in the geopolymeric lattice, which have greater strength, contributing to higher geopolymer structural integrity.^40,56^ Increasing the SiO_2_/Al_2_O_3_ ratio from 2.74 to 3.50 at a fixed Al_2_O_3_/Na_2_O ratio of 1.3 (mix #3 and 23, respectively), the compressive strength increased by 640%. Further increase in the SiO_2_/Al_2_O_3_ ratio in the geopolymeric matrix saturates the bulk solution with a massive amount of SiO_2_, preventing the metakaolin dissolution.^40,49^ It can be concluded that the optimum SiO_2_/Al_2_O_3_ ratio was approximately 3.5. This result agrees with those of the previous studies.^39,40,49^ For instance, Duxson *et al.*^39^ reported that the compressive strength of metakaolin-based geopolymers increased linearly by about 400% as the SiO_2_/Al_2_O_3_ ratio increased from 3.3 to 3.8, reaching its maximum value before decreasing again at the highest ratio of 4.3.

At the optimum SiO_2_/Al_2_O_3_ ratio of 3.5, the compressive strength exhibited significant variations (from 3.04 to 50.80 MPa) as indicated in Table 3. This clarifies that the Al_2_O_3_/Na_2_O ratio plays an important role in determining the compressive strength of metakaolin-based geopolymers. Most of the previous studies have focused on studying the Al_2_O_3_/Na_2_O ratio effect in the range of 1. Theoretically, Al_2_O_3_/Na_2_O = 1 represents the stoichiometry for neutralizing the negative charge present on [Al(OH)_4_]^−^, which results from the dissolution of Al_2_O_3_.^40,49,68^ Duxson *et al.*^39^ and Stevenson and Sagoe-Crentsil^69^ reported that higher strength values were recorded when the SiO_2_/Al_2_O_3_ and Al_2_O_3_/Na_2_O ratios were 3.00–3.80 and about 1, respectively. However, they did not study the influence of increasing the Al_2_O_3_/Na_2_O ratio to values higher than 1 with a constant H_2_O/Na_2_O ratio. Therefore, to examine the effect of a wide range of Al_2_O_3_/Na_2_O ratios on the compressive strength of geopolymer samples, the mixes were formulated with a ratio ranging from 0.75 to 1.50. It can be observed that as the Al_2_O_3_/Na_2_O ratio increased, the compressive strength significantly enhanced. At a SiO_2_/Al_2_O_3_ ratio of 3.50, increasing the Al_2_O_3_/Na_2_O ratio from 0.85 to 1.40 enhanced the compressive strength by 1570%. Additionally, from Table 3 and the contour plot (Fig. 4), it can be seen that at high SiO_2_/Al_2_O_3_ ratios (3.90, 4.10 and 4.30), the specimens with high Al_2_O_3_/Na_2_O cannot be mixed, as discussed above. At SiO_2_/Al_2_O_3_ ratios of 3.1, 3.3, 3.5, 3.7, 3.9, 4.1 and 4.3, the highest strength values were achieved at an Al_2_O_3_/Na_2_O ratio of 1.50, 1.45, 1.40, 1.30, 1.00, 0.85 and 0.75, respectively. Similar findings were reported in previous studies. Ghanbari *et al.*^34^ achieved the highest compressive strength of 65.9 MPa in a metakaolin-based geopolymer with SiO_2_/Al_2_O_3_ and Al_2_O_3_/Na_2_O ratios of 2.9 and 1.72, respectively. Kong *et al.*^45^ achieved 45 MPa with ratios of 3.08 and 1.52. Barbosa *et al.*^48^ reached 49 MPa with ratios of 3.3 and 1.2. Also, Duxson *et al.*^39^ reported the highest strength at 82 MPa with ratios of 3.8 and 1.0, while Lahotia *et al.*^33^ attained 72.5 MPa with ratios of 4.0 and 0.91. Rowles and O'Connor^38^ reported 64 MPa with ratios of 5.0 and 0.78, respectively. This indicates that SiO_2_/Al_2_O_3_ and Al_2_O_3_/Na_2_O ratios control the kinetics of formation of the three-dimensional geopolymeric structure. Generally, the Na_2_O in geopolymer reactions has two main roles. Firstly, it acts as an alkaline activator that must be present in sufficient quantities (expressed in the alkalinity of the activator solution) to allow dissolution of the aluminosilicate precursors.^70^ Secondly, it is used to enable charge balance between the alkali cation and the tetrahedrally coordinated aluminum, as illustrated above. According to the experimental results of this study, as shown in Table 3, increasing the Al_2_O_3_/Na_2_O ratio to 1.50, depending on the SiO_2_/Al_2_O_3_ ratio, enhances the compressive strength. This discrepancy may occur due to several practical factors: (i) not all the alumina in the aluminosilicate structure (metakaolin) is reactive, hindering their dissolution; this depends on structure constraints, such as the quantity of residual crystalline phase, as described by Scherb *et al.*^71^ This is supported by the XRD-pattern of metakaolin (Fig. 2); therefore, low Na_2_O content is required. (ii) Excess Na_2_O, which refers to the presence of a high amount of Na^+^ ions beyond the required amount, disturbing the geopolymer skeleton.^68^ (iii) While maintaining SiO_2_/Al_2_O_3_ and H_2_O/Na_2_O ratios, it is observed that increasing Al_2_O_3_/Na_2_O is accompanied by decreasing water/solid ratio, denoting the formation of dense structures with high mechanical performance.^72^ According to the previous results, it can be concluded that the geopolymerization process cannot be interpreted by considering the Al_2_O_3_/Na_2_O molar ratio independently from the SiO_2_/Al_2_O_3_ molar ratio. The Al_2_O_3_/Na_2_O ratio of 1 cannot be regarded as a universal optimum. Rather, it represents a theoretical stoichiometric condition for charge balancing of negatively charged AlO_4_^−^ units within the geopolymer structure under ideal assumptions, such as full availability and integration of Al into the geopolymeric network. To confirm these arguments, qualitative and quantitative investigations were performed using XRD and TGA/DTG, as well as the SEM and BET methods to detect the morphology of hydration products inside the pores.

Finally, it can be reported that the compressive strength of the metakaolin geopolymers is strongly affected by SiO_2_/Al_2_O_3_, Al_2_O_3_/Na_2_O, Na_2_O availability, and water/solid ratio. From the result illustrated above, it was found that the main factors controlling the compressive strength of the geopolymer are the SiO_2_/Al_2_O_3_ molar ratio, followed by the Al_2_O_3_/Na_2_O molar ratio. Moreover, to determine which Al_2_O_3_/Na_2_O or water/solid ratio is considered the most powerful factor that significantly contributes to improving the compressive strength, further investigation was required. From mix #1–32, changing the Al_2_O_3_/Na_2_O ratio across mixes at a fixed SiO_2_/Al_2_O_3_ ratio requires changing the amount of Na_2_O. Simultaneously, to maintain the H_2_O/Na_2_O ratio at 9.5 across all mixes, the amount of water used to prepare the alkaline solution must be changed; accordingly, the water/solid ratio changes. This coupling must be considered when interpreting the strength results. For instance, at a SiO_2_/Al_2_O_3_ ratio of 3.50, increasing the Al_2_O_3_/Na_2_O ratio from 0.85 to 1.40 was accompanied by a decrease in the water/solid ratio from 0.49 to 0.32, while the compressive strength increased from 3.04 to 50.80 MPa. This indicates that the enhancement in the strength cannot be attributed exclusively to Al_2_O_3_/Na_2_O. The clarification of the relative contribution of Al_2_O_3_/Na_2_O and the water/solid ratio in the compressive strength was performed by preparing and testing two supplementary mixes (mix #33 and 34). For the specimens with a SiO_2_/Al_2_O_3_ ratio of 3.5, mix #33 was prepared with Al_2_O_3_/Na_2_O of 1.3 and water/solid of 0.43 to be compared with mix #20 with the same water/solid ratio and different Al_2_O_3_/Na_2_O (18.56 MPa) and mix #23 with the same Al_2_O_3_/Na_2_O and different water/solid ratio (48.48 MPa). It was found that mix #33 has a compressive strength of 41.57 MPa. This indicates that, at a constant water/solid ratio of 0.43 and at different Al_2_O_3_/Na_2_O ratios of 1.0 and 1.3, as in mix #20 and mix #33, respectively, the compressive strength of mix #33 is higher than that of mix #20 by 124.0%. This demonstrates that increasing Al_2_O_3_/Na_2_O has a pronounced effect on the strength, even when the water/solid ratio is maintained constant. On the other hand, at a constant Al_2_O_3_/Na_2_O of 1.3 and different water/solid ratios of 0.34 and 0.43, as in mix #23 and mix #33, respectively, the compressive strength of mix #23 is higher than that of mix #33 by 16.6%. This confirms that the reduction in the water/solid ratio contributes to improving the strength by promoting a denser microstructure. However, the significant increase observed between mix #20 and mix #33 shows that the enhancement in strength is not governed by water/solid ratio alone. A similar trend was observed for the specimens with a SiO_2_/Al_2_O_3_ ratio of 3.7, mix #34 was prepared with Al_2_O_3_/Na_2_O of 1.3 and water/solid of 0.41 to be compared with mix #26 with the same water/solid ratio and different Al_2_O_3_/Na_2_O (31.92 MPa) and mix #28, with the same Al_2_O_3_/Na_2_O and different water/solid ratio (43.20 MPa). It was found that mix #34 has a compressive strength of 40.84 MPa. This indicates that, at a constant water/solid ratio of 0.41 and at different Al_2_O_3_/Na_2_O ratios of 1.0 and 1.3, as in mix #26 and mix #34, respectively, the compressive strength of mix #34 is higher than that of mix #26 by 27.9%. Conversely, at a constant Al_2_O_3_/Na_2_O ratio of 1.3 and different water/solid ratios of 0.33 and 0.41, as in mix #28 and mix #34, respectively, the compressive strength of mix #28 is higher than that of mix #34 by 5.8%. Furthermore, a semi-quantitative attribution supports this interpretation. At a SiO_2_/Al_2_O_3_ ratio of 3.5, the strength increased by 29.92 MPa from mix #20 to mix #23. When the water/solid ratio was fixed at 0.43 (mix #33), 23.01 MPa of this gain was preserved, corresponding to 76.9% of the total strength development. At SiO_2_/Al_2_O_3_ = 3.70, 79.1% of the strength increase from mix #26 to mix #28 was preserved in mix #34 after adjusting the water/solid ratio to 0.41. These results indicate that, in the selected comparisons, the Al_2_O_3_/Na_2_O ratio has a stronger influence on compressive strength than the water/solid ratio, although both variables contribute. This can be attributed to the role of Na_2_O in controlling alkalinity, facilitating metakaolin dissolution, compensating charges of AlO_4_^−^ units, and forming a continuous aluminosilicate network. However, the water/solid ratio remains crucial because it influences the densification of the microstructure. This is also consistent with the conclusions of Lahoti *et al.*^33^ They found that the water/solid ratio is the least influential factor on the geopolymer's compressive strength compared to other mix-design parameters.

#### 3.3.2. Statistical analysis

To understand the key factors influencing the properties of geopolymers (molar ratios of SiO_2_/Al_2_O_3_ and Al_2_O_3_/Na_2_O), a statistical study was performed using ANOVA. The calculated results from the ANOVA (*F*-values and *p*-values) were considered statistically significant for the response factor at a 95% confidence level. The relative relevance of each factor was assessed using the *F*-values; *F* > *F*_crit_ indicates a significant impact of the factor. Furthermore, when the *p*-value is lower than a critical level of *a* = 0.05, it also implies the statistical significance of the factor. The ANOVA analysis results in Table 4 show that, for all the studied factors, *F* > *F*_crit_ and *p*-value < 0.05, indicating that all the examined parameters were statistically significant.^20,63^

**Table 4:** ANOVA results for the compressive strength after 28 days for selected specimens

Factor	Condition	SS		Df		MS		*F*	*P*-value	*F*_crit_	Significance criteria (*P* < 0.05)
		Between groups	Within groups	Between groups	Within groups	Between groups	Within groups				
Effect of different SiO_2_/Al_2_O_3_ with constant Al_2_O_3_/Na_2_O = 1.3	Compressive strength for mixes# 3, 6, 9, 16, 23 and 28	3578	32	5	12	715.55	2.67	267.72	1.1 × 10^−11^	3.11	Significant
Effect of different Al_2_O_3_/Na_2_O with constant SiO_2_/Al_2_O_3_ = 2.74	Compressive strength for mixes 1, 2 and 3	104	0.12	2	6	51.925	0.02	2704.40	3.5 × 10^−11^	5.14	Significant
Effect of different Al_2_O_3_/Na_2_O with constant SiO_2_/Al_2_O_3_ = 2.9	Compressive strength for mixes 4, 5 and 6	118	0.74	2	6	59.085	0.12	479.19	1 × 10^−7^	5.14	Significant
Effect of different Al_2_O_3_/Na_2_O with constant SiO_2_/Al_2_O_3_ = 3.5	Compressive strength for mixes 19, 20, 21, 22, 23 and 24	1911	36.71	5	12	382.185	3.06	124.94	1.6 × 10^−7^	3.11	Significant
Effect of different Al_2_O_3_/Na_2_O with constant SiO_2_/Al_2_O_3_ = 3.7	Compressive strength for mixes 25, 26, 27 and 28	4259	54.07	3	8	1419.72	6.76	210.07	3 × 10^−8^	4.07	Significant

### 3.4. Phase composition and microstructure analysis

In this study, some specimens were selected to examine the impact of various factors affecting the preparation of the mix-design on the phase composition and of the prepared metakaolin geopolymer. The selection of these specimens was based on examining representative mixes that covered different framework compositions and strength levels within the experimental matrix. Mix #11 represents a mix with low SiO_2_/Al_2_O_3_ composition and high Al_2_O_3_/Na_2_O. Mix #19 signified the low-strength mixture at SiO_2_/Al_2_O_3_ of 3.50 and low Al_2_O_3_/Na_2_O. Mix #24 represented the highest-strength mixture in the experimental matrix; mix #31 represented a higher SiO_2_/Al_2_O_3_ composition. In mixes #11 and 24, the H_2_O/Na_2_O and water/solid ratio are similar, with a very small difference in Al_2_O_3_/Na_2_O; this allows measuring the impact of SiO_2_/Al_2_O_3_ on the phase composition, texture characteristics and microstructure. In mixes #19 and 31, the Al_2_O_3_/Na_2_O and H_2_O/Na_2_O ratios are similar; this also permits investigating the impact of SiO_2_/Al_2_O_3_ and water/solid ratio. In mixes #19 and 24, the SiO_2_/Al_2_O_3_ and H_2_O/Na_2_O ratios are similar; this also enables examining the impact of Al_2_O_3_/Na_2_O and water/solid ratio. Because the different analysis techniques (XRD, FTIR, TGA/DTG, SEM/EDX and BET/BJH) were applied only to a select set of representative mixtures, the findings regarding phase composition and microstructure are considered supportive evidence for the overall relationship between the mixtures' composition and strength rather than a complete structural verification of all thirty-four mixtures.

#### 3.4.1. X-ray diffraction

**Fig. 5:**
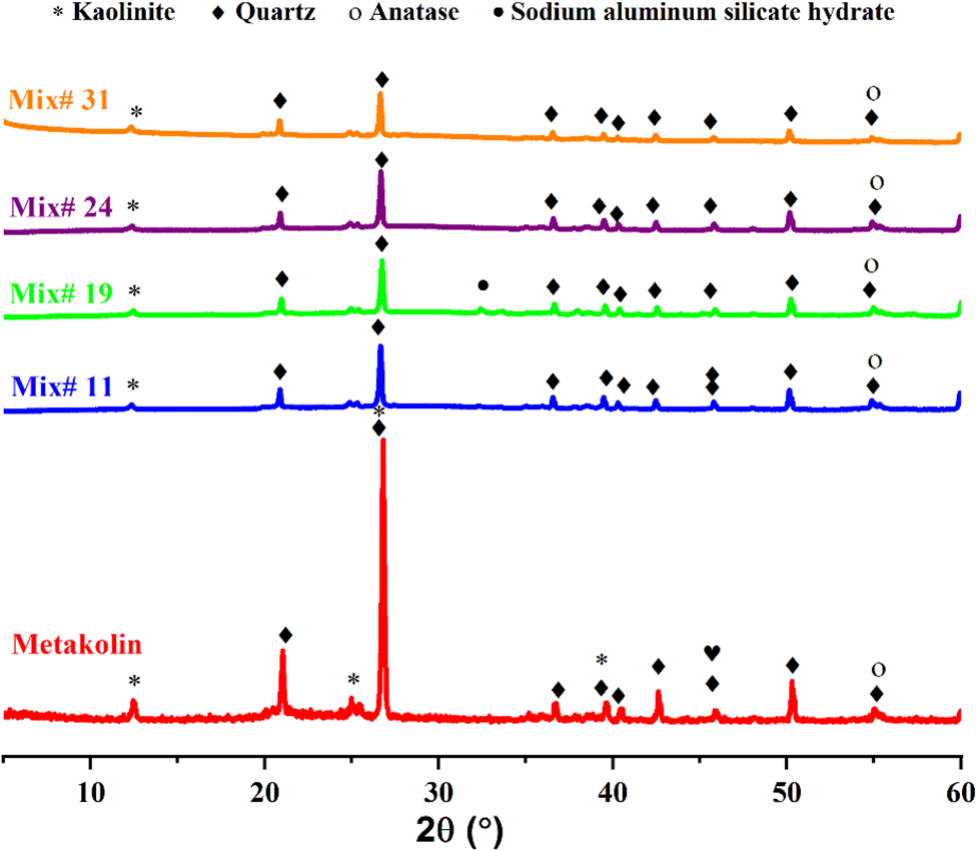
XRD patterns of metakaolin and selected metakaolin geopolymer after 28 days.

The impact of alkaline activation on the phase composition of metakaolin was examined using XRD analysis, as shown in Fig. 5. Also, some specimens were selected to be analyzed to investigate the impact of SiO_2_/Al_2_O_3_ and Al_2_O_3_/Na_2_O molar ratios on the phase composition of the metakaolin geopolymer. The specimens were mix #11, 19, 24 and 31 with SiO_2_/Al_2_O_3_ ratios of 3.1, 3.5, 3.5 and 4.1 and Al_2_O_3_/Na_2_O ratios of 1.50, 0.85, 1.40 and 0.85, respectively. As described above in Fig. 2, the metakaolin is composed of a small quantity of kaolinite, in addition to quartz and anatase phases, indicating that they are mostly inactive. After alkali activation, the reflection peaks of these phases are still present in the metakaolin geopolymer with a significant reduction in their intensities.^73^ Moreover, the broad band observed in metakaolin at 2*θ* = 24°–26° becomes weaker or disappears in geopolymer specimens, indicating the dissolution of the amorphous aluminosilicate precursor and the subsequent formation of a strength-giving phase gel (sodium–alumino–silicate–hydrate, NASH), as seen at 2*θ* = 32.41°. This aligns with reports by Dai *et al.*^74^

By comparing the XRD pattern of mix #11 and 24, which have similar Al_2_O_3_/Na_2_O (1.50 and 1.40, respectively), H_2_O/Na_2_O (9.50) and water/solids ratio (0.32), but different SiO_2_/Al_2_O_3_ ratio (3.1 and 3.5, respectively), it can be detected that mix #24 displays the lowest intensity of unreacted phases. This reflects the role of sufficient SiO_2_/Al_2_O_3_ in the dissolution of inactive phases. Increasing the SiO_2_/Al_2_O_3_ ratio to 3.5 (containing a high quantity of active silica) forms reactive monomers (Si–OH) and less-ordered oligomers (Si–O–Si). These active species directly attack Si- and Al-sites on the surface of the metakaolin, break Al–O and Si–O bonds, and dissolve the aluminosilicate structure, as discussed by Gao *et al.*^35^ Therefore, the leaching of Si and Al may be the reason behind the decrease in the peak intensity of inactive phases in metakaolin and the formation of strength-giving-phases, correlating with its superior mechanical performance (50.8 MPa).

On the other hand, the XRD patterns of both mix #19 and 24 specimens, which have the same SiO_2_/Al_2_O_3_ (3.5) and H_2_O/Na_2_O (9.50) ratios but differ in Al_2_O_3_/Na_2_O (0.85 and 1.40, respectively) and water/solids (0.49 and 0.32, respectively) ratios, show approximately the same intensity for the present phases. Rather than a lower Al_2_O_3_/Na_2_O (high Na_2_O content), as in mix #19, which can disturb the charge balancing in the geopolymer chains, it also causes pore coarsening,^75,76^ as will be described below in the discussion on texture/microstructure analysis. Furthermore, the high water/solid ratio in mix #19 (0.49) compared to mix #24 (0.32) increases the porosity, weakening the matrix. These factors likely explain the reduction in compressive strength from 50.80 MPa (mix #24) to 3.04 MPa (mix #19). The same reasons may also account for the low compressive strength of mix #31 compared to mix #24.

Generally, XRD is considered a qualitative/semi-quantitative analysis technique. The Rietveld refinement was performed using Match! 3 software for metakaolin raw material and selected geopolymer specimens (mix #11, 19, 24 and 31) to provide quantitative support for the XRD interpretation, as shown in Table 5. As the main binding phases in geopolymer are amorphous or poorly crystalline (NASH), the Rietveld refinement is used primarily to quantify the residual crystalline precursor-related phases. The amount of NASH is estimated below using the TGA/DTG analysis. Table 5 shows that the raw metakaolin contains 30.2% kaolinite, 68.3% quartz and 1.5% anatase. After alkaline activation, the kaolinite phase decreased across all the tested geopolymeric mixes, confirming that a portion of the aluminosilicate precursor was dissolved and converted into geopolymeric products. The residual kaolinite phase decreased from 30.2% in the raw material to 19.7%, 19.2%, 18.6% and 23.7% in mixes #11, 19, 24, and 31, with reductions of 34.8%, 36.4%, 38.4% and 21.5%, respectively.

**Table 5:** Quantitative phase analysis of raw metakaolin and selected geopolymer specimens

Mix no.	Kaolinite	Quartz	Anatase
Metakaolin raw material	30.2	68.3	1.5
Mix #11	19.7	78.8	1.5
Mix #19	19.2	79.3	1.5
Mix #24	18.6	79.8	1.6
Mix #31	23.7	74.6	1.7

It is worth noticing that mix #24 shows the lowest amount of kaolinite phase, indicating it has a favorable mix design, which facilitates the dissolution of metakaolin and the formation of geopolymer products. This behavior is consistent with its balanced starting composition, which provides enough active silica for the development of a geopolymer network, sufficient Na^+^ ions for charge balancing of AlO_4_^−^ and a limited amount of excess water that densifies the microstructure.^40,49,56,68^ On the other hand, the higher kaolinite phase in mix #11, with respect to mix #24, can be attributed to the limited availability of active silicate needed for developing a highly connected aluminosilicate network.^40^ This will be proved below by the SEM, which shows the mix #11 matrix contains unreacted particles of metakaolin engulfed in a heterogeneous microstructure. Mix #19 has a kaolinite phase close to that of mix #11, confirming that an adequate amount of metakaolin was participating in the geopolymerization process. Despite this observation, it was found that mix #19 has a lower compressive strength than mix #11. Accordingly, this reduction in the strength values can be attributed to the excess Na^+^ ions, which can disturb the geopolymer network.^68^ Also, the high water content may be another reason behind its low compressive strength. Mix #31 shows the highest residual kaolinite phase content among the analyzed geopolymeric mixes, 23.7%. This proves that the excess silicate, excess Na^+^ ions and high water/solid ratio hinder the dissolution of metakaolin and promote heterogeneous precipitation, as illustrated above.^40,49,68^

The quartz content increased from 68.3% in the raw metakaolin to 78.8%, 79.3%, 79.8% and 74.6% in mixes #11, 19, 24 and 31, respectively. Generally, the quartz is relatively inert under the highly alkaline medium.^77^ Accordingly, it can be discussed that the increase in quartz in geopolymers relative to raw metakaolin is due to a decrease in the reactive kaolinite phase; therefore, mix #24 has the highest quartz content. Also, the anatase phase was nearly stable across all specimens, which ranged from 1.5% to 1.7%. This demonstrates that anatase did not participate in the geopolymerization reaction and instead behaved as a stable minor crystalline impurity.^78,79^

#### 3.4.2. FTIR analysis

**Fig. 6:**
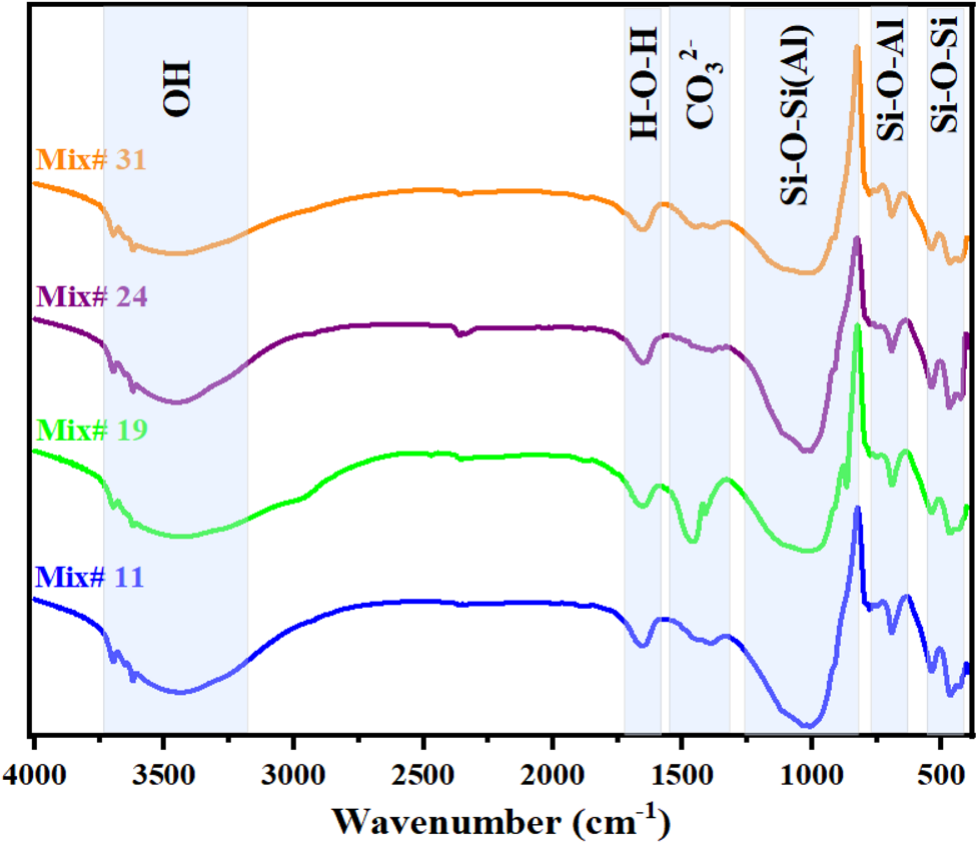
FTIR spectra of metakaolin and selected metakaolin geopolymer after 28 days.

Regarding the development of the metakaolin geopolymerization process with changing SiO_2_/Al_2_O_3_ and Al_2_O_3_/Na_2_O molar ratios, the functional groups were analyzed using the FTIR technique. The FTIR spectra for mixes #11, 19, 24 and 31 are represented in Fig. 6. The spectra for all specimens show regular features for metakaolin geopolymers. The broad band in the range of 3060–3770 cm^−1^ and another in the range of 1570–1750 cm^−1^ are associated with the OH symmetric stretching and H–O–H vibration bending, respectively. The existence of these functional groups arises from physically/chemically-combined water and hydroxyl groups in the geopolymeric gel.^80^ Furthermore, the intensity of these bands is affected by the water/solid ratio and the extent of the geopolymerization process.^81^ In mixes with a high water/solid ratio, these bands are generally more pronounced. Mix #19 shows a relatively high H–O–H, which is expected due to its high water/solid ratio of 0.49. Also, it verified its low compressive-strength value (3.04 MPa), as the strong H–O–H related band refers to retained pore water and less condensed gel. Mix #31 also shows noticeable O–H and H–O–H bands, aligning with its relatively high water/solid ratio of 0.45 and its strength of 21.49 MPa, which is higher than that of mix #19. Mix #24, which has the highest strength value of 50.8 MPa, shows a pronounced H–O–H band but is less dominant relative to the main geopolymer Si–O–Si(Al) network region. This indicates that the retained water is correlated with gel water rather than excessive free pore water, supporting the formation of a more condensed aluminosilicate network and then a denser microstructure. Regarding mix #11, the H–O–H related band cannot be used as an indication for its relatively low strength of 21.49 MPa because it essentially started with a low water/solid ratio of 0.32.

The band in the region around 1360–1560 cm^−1^ can be correlated with CO_3_^2−^ stretching.^82^ Generally, the carbonate species are produced by carbonation of the residual alkalis with atmospheric CO_2_. Carbonate bands are frequently observed in alkali-activated systems, particularly when there is an excess of alkali present.^83^ This suggests that excess Na_2_O is not necessary to enhance activation but might lead to secondary products that do not significantly strengthen the material.

The key structural region that characterizes the formation of the geopolymer is found in the range of 825–1300 cm^−1^, which is assigned to Si–O–Si(Al) asymmetric stretching. This band is considered the fingerprint of the formation of the aluminosilicate geopolymer network.^84^ Interestingly, mix #24 exhibits the strongest and most well-developed Si–O–Si(Al) band among the selected compositions. When comparing mix #19, which has a compressive strength of 3.04 MPa, with mix #24, which has a strength value of 50.80 MPa, it is notable that both mixes have the same SiO_2_/Al_2_O_3_ ratio of 3.5. However, mix #24 has a higher Al_2_O_3_/Na_2_O ratio and a lower water/solids ratio. This proves that the improvement in the compressive strength cannot be attributed to the presence of active silica alone, but it can be explained by the combined effect of sufficient active silica, Na^+^ ion availability for balancing the AlO_4_^−^ negative charge and low water/solid ratio.^40,49,68,75^ These data confirm that mix #24 is composed of an activator with enough alkalinity for metakaolin dissolution without excessive Na^+^ free ions and water, causing the formation of connected and denser N–A–S–H. On the contrary, for mix #19, which has a relatively high Na_2_O content (low Al_2_O_3_/Na_2_O of 0.85) and a high water/solid ratio of 0.49 compared with mix #24, the FTIR spectrum shows a less favorable geopolymeric structure. This conclusion was drawn from noticing increased contributions from H–O–H and CO_3_^2−^ bands, as well as a less optimized Si–O–Si(Al) network. Although relatively excess Na^+^ ions and high water/solid ratio can initially enhance the dissolution of metakaolin, they can disturb the formation of the geopolymer network, increasing the porosity and promoting the formation of carbonate phases through the reaction with atmospheric CO_2_.^75,76^ Mix #11 exhibits an Si–O–Si(Al) band but with a lower compressive strength of 12.56 MPa, compared with mix #24. The lower SiO_2_/Al_2_O_3_ (3.10) and high Al_2_O_3_/Na_2_O (1.50) in mix #11 indicate the insufficient availability of Na_2_O to fully activate and charge balance the aluminate species. The FTIR spectrum indicates partial formation of a geopolymer gel, but it does not suggest the creation of a highly continuous and mechanically effective network. Mix #31 has the highest SiO_2_/Al_2_O_3_ ratio of 4.10, revealing a silica-rich formulation. The FTIR spectrum shows pronounced silicate-related bands, implying an increased Si–O–Si contribution. As illustrated above, the excess SiO_2_ content can saturate the bulk solution, hindering the dissolution of metakaolin.^40,49^ Furthermore, this mix is characterized by low Al_2_O_3_/Na_2_O (0.85) and high water/solid ratio (0.45); therefore, excess soluble SiO_2_, free Na^+^ ions, and high water content may lead to a less homogeneous gel. This explains why mix #31 reaches only 21.49 MPa. The low-wavenumber region below 820 cm^−1^ provides further data related to the formation of the geopolymeric gel network. The band in the range of 635–725 cm^−1^ and another one in the range of 400–500 cm^−1^ are accompanied by the Si–O–Al symmetric stretching and Si–O–Si bending, respectively. These bands also contribute to the formation of an aluminosilicate gel network.^80^

#### 3.4.3. Thermal analysis

**Fig. 7:**
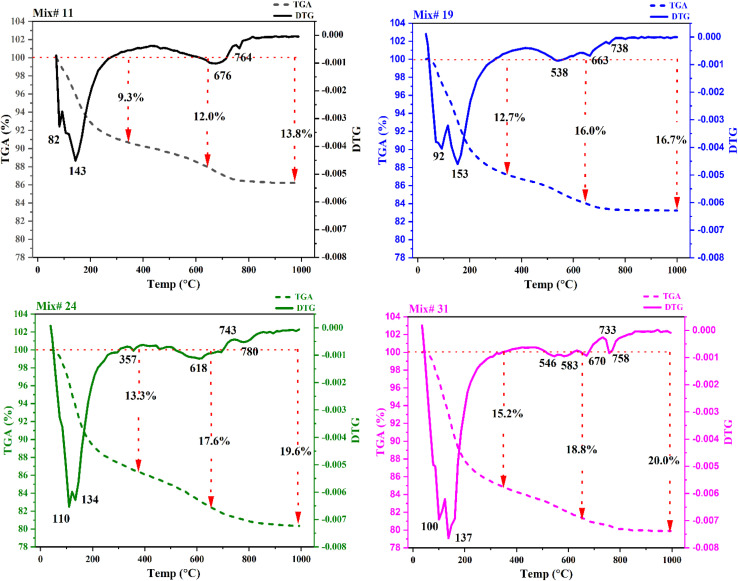
TGA/DTG curves of selected metakaolin geopolymers after 28 days.

Four geopolymer specimens (mix #11, 19, 24 and 31) were selected for qualitative and quantitative analyses of existing phases at 28 days of casting using thermal analysis techniques (TGA/DTG), as shown in Fig. 7. The analysis clarified that all the geopolymer specimens exhibit a sharp mass reduction within the temperature range from ambient temperature to 200 °C, reaching 7.1%, 9.8%, 10.7% and 12.3% and accounting for about 51%, 59%, 55% and 61% of the total mass loss for mix #11, 24, 19 and 31, respectively. These mass losses are accompanied by two endothermic peaks centered at 82 °C and 140 °C, 92 °C and 153 °C, 110 °C and 134 °C, as well as 100 °C and 137 °C, respectively. These are presumed to be due to the evaporation of both free water and a part of the tightly bound water from the geopolymer structure.^85–87^ The water/solid ratio for both mix #11 and 24 (high Al_2_O_3_/Na_2_O = 1.40–1.50) was 0.32, while it was 0.49 and 0.45 in mix #19 and 31 (low Al_2_O_3_/Na_2_O = 0.85), respectively. This explains the significant mass-loss% accompanied by heating up to 200 °C, for both mix #19 and 31, compared with mix #11 and 24. It is noteworthy that the endothermic second peak below 200 °C for mix #24 shifted slightly to a higher temperature (153.2 °C) than the other mixes. This proves that increasing both SiO_2_/Al_2_O_3_ and Al_2_O_3_/Na_2_O ratios up to 3.5 and 1.4, respectively, makes it difficult for water to evaporate. This indicates that this mix design (mix #24) reduced the void ratio and formed a tighter bond within the geopolymer gel, producing a dense matrix. This is consistent with the compressive strength achieved by this sample, which reached 50.80 MPa (Table 3). When the Al_2_O_3_/Na_2_O ratio decreased to 0.85 in mix #19 and 31, the endothermic peaks became steeper and shifted to lower temperatures at 133.9 °C and 136.7 °C, respectively. This is mainly due to the fact that the large excess amounts of water lead to an increase in the porosity in the matrix and decrease the ability of the sample to retain water.^88^ This is consistent with the reduced values of compressive strengths of mix #19 compared to 24 (which have the same SiO_2_/Al_2_O_3_ ratio) as a result of the decrease in the Al_2_O_3_/Na_2_O ratio, where it decreased from 50.80 to 3.04 MPa, respectively (Table 3).

The geopolymers continue to lose weight until 350 °C, with a total loss of about 9.3%, 12.7%, 13.3% and 15.2% for mix #11, 19, 24 and 31, respectively. This might be induced by the dehydroxylation process of geopolymers and a small quantity of kaolinite phase present in metakaolin.^70,89,90^ This dehydroxylation process continues up to 650 °C with further mass-loss% of approximately 2.9%, 3.3%, 4.3% and 3.6%, respectively.^91,92^ The high mass-loss% for mix #24 with respect to mix #11 and 19 refers to the roles of optimum SiO_2_/Al_2_O_3_ and Al_2_O_3_/Na_2_O ratios (3.5 and 1.4, respectively) in enhancing the hydraulic nature and thermal stability of the formed geopolymeric phases, matching its superior mechanical characteristics. The slightly high mass-loss% of mix #31 with respect to mix #24 is due to the water/solid ratio, as illustrated above. Furthermore, these mass-losses% may also be due to the elimination of water, which resulted from condensation of silanol (Si–OH) or aluminol (Al–OH) groups on the surface of the geopolymer gel.^85^ Finally, the mass-loss% associated with several endothermic peaks at higher temperatures beyond 650 °C, reaching 1.6%, 0.7%, 2% and 1.2%, for mix #11, 19, 24 and 31, may be correlated to the decomposition of carbonated phases, resulting from exposure of the specimens to the CO_2_ from air.^10,93^

#### 3.4.4. Scanning-electron microscopy

**Fig. 8:**
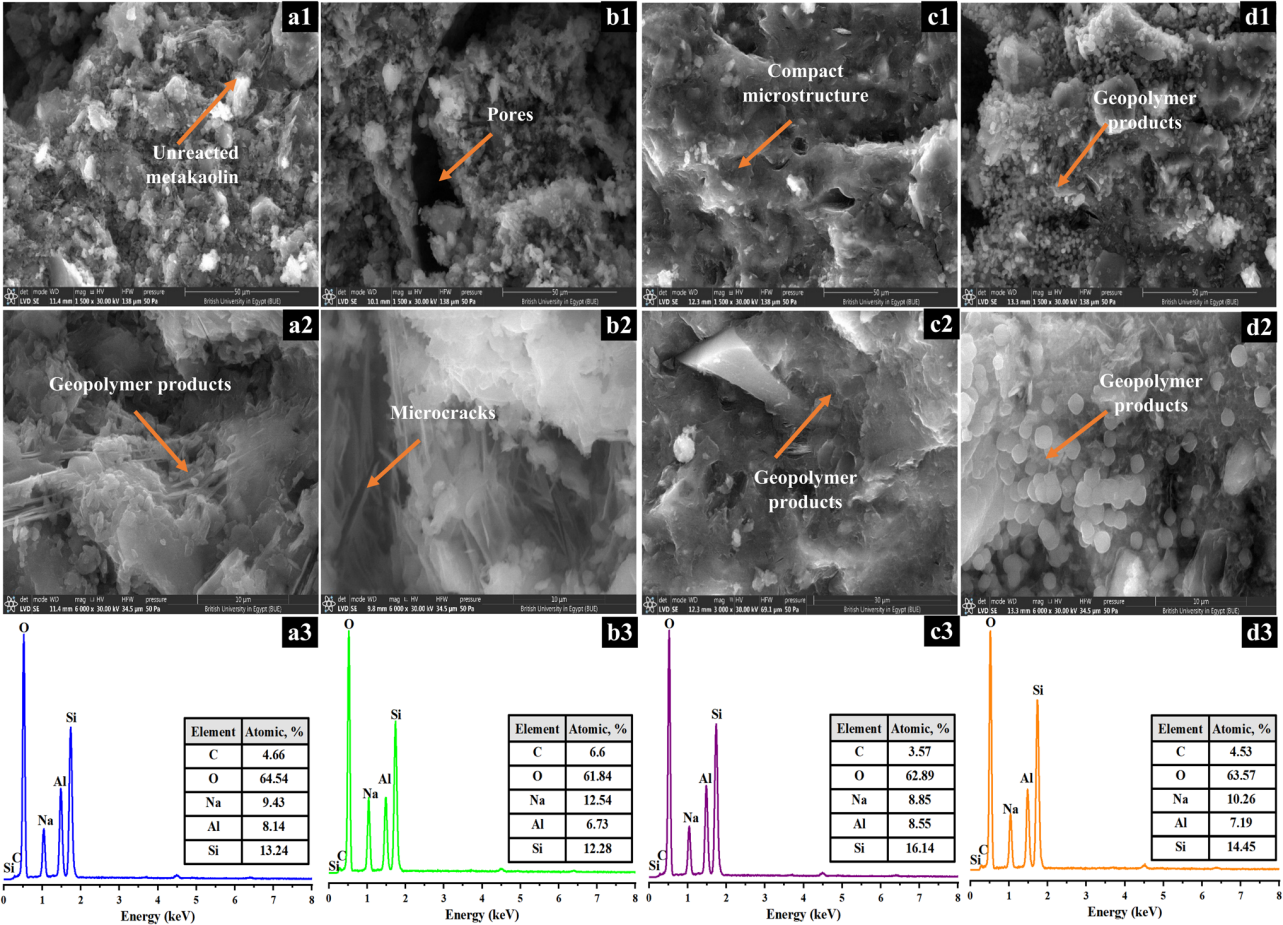
SEM/EDX images and spectra of selected metakaolin geopolymer after 28 days: (a1–a3) mix #11, (b1–b3) mix #19, (c1–c3) mix #24 and (d1–d3) mix #31.

After 28 days of hydration, SEM/EDX analysis was conducted on mixes #11, 19, 24 and 31 to clarify the relationship between mix design (SiO_2_/Al_2_O_3_ and Al_2_O_3_/Na_2_O molar ratio), microstructure, elemental composition and compressive strength. SEM was used to follow up on the microstructure development and morphology of hydration products. The full-area EDX analysis provided a semi-quantitative estimate of the regional average elemental composition (at%). These data may be relevant for the current study, as the mechanical performance of the produced metakaolin geopolymer depends on the overall chemistry and uniformity of the hardened matrix. Fig. 8 shows that these molar ratios have a significant impact on the characteristics of the geopolymeric matrix. Also, the EDX analysis shows the presence of Si, Al, Na, C and O elements, matching the chemical composition of metakaolin and the formed strength-giving phases (NASH). Since the initial SiO_2_/Al_2_O_3_ and Al_2_O_3_/Na_2_O ratios are based on oxide molar ratios, and EDX provides elemental (at%) data, the starting SiO_2_/Al_2_O_3_ in the mix design was divided by 2 to convert it to Si/Al, while Al_2_O_3_/Na_2_O corresponds to Al/Na, as shown in Table 6. Interestingly, noticing that the EDX Si/Al ratio and Al/Na ratios are in line with the starting SiO_2_/Al_2_O_3_ ratios. The starting Si/Al ratios in the mix design were 1.55, 1.75, 1.75 and 2.05, while in the EDX analysis were 1.63, 1.82, 1.89 and 2.01 for mixes #11, 19, 24 and 31, respectively. However, the EDX analysis of the Al/Na ratios was higher than the theoretical starting Al/Na ratios. The starting Al/Na in the mix design were 1.50, 0.85, 1.40 and 0.85, while in the EDX analysis were 0.86, 0.53, 0.97 and 0.70 for mixes #11, 19, 24 and 31, respectively. This difference can be attributed to the high mobility of Na^+^ ions and the possibility of their accumulation near pores/surfaces or forming secondary sodium-rich products. Wang *et al.*^94^ and Zhang *et al.*^95^ demonstrated that Na^+^ ions in the geopolymer are trapped within the pores of the surface because of their strong interaction with surface oxygen. Beltrame *et al.*^96^ observed that sodium carbonate crystals can form inside pores due to carbonation of Na-rich materials. Jiang *et al.*^97^ found that in metakaolin-based geopolymers, about 52% of alkali ions are weakly bound, while the remaining amount is stabilized within the material.

**Table 6:** EDX analysis of selected specimens

Mix no.	Starting SiO_2_/Al_2_O_3_	Starting elemental Si/Al	Starting Al_2_O_3_/Na_2_O	Starting elemental Al/Na	EDX Si/Al	EDX Al/Na
Mix #11	3.10	1.55	1.50	1.50	1.63	0.86
Mix #19	3.50	1.75	0.85	0.85	1.82	0.53
Mix #24	3.50	1.75	1.40	1.40	1.89	0.97
Mix #31	4.10	2.05	0.85	0.85	2.01	0.70

In mix #11, which has SiO_2_/Al_2_O_3_, Al_2_O_3_/Na_2_O, and water/solid ratios of 3.1, 1.50, and 0.32, respectively, the SEM images reveal a heterogeneous microstructure (Fig. 8a1 and 2). This microstructure contains lumps of platy grains that likely consist of unreacted metakaolin particles. This observation suggests that the metakaolin particles have partially dissolved, leading to disruptions in the aluminosilicate network connections.^20^ The EDX analysis (Fig. 8a3) shows the presence of Si, Al and Na, confirming the formation of the geopolymeric products. Table 6 demonstrates that the EDX Si/Al ratio of mix #11 is lower than those of mixes #19, 24 and 31. Accordingly, a moderate amount of silica in the geopolymeric matrix, despite the presence of sodium, limits the formation of a three-dimensional network structure, resulting in an inadequate compressive strength of 12.56 MPa.

For mix #19, although it has a high SiO_2_/Al_2_O_3_ ratio (3.5) compared to mix #11 (3.1), the compressive strength decreased by 75.79%. The SEM images (Fig. 8b1 and 2) represent the formation of a porous matrix containing microcracks. EDX analysis in Fig. 8b3 and Table 6 reveals that mix #19 has a Si/Al ratio of 1.82 and the lowest Al/Na ratio of 0.53. The Si/Al ratio implies that the geopolymeric matrix contains sufficient silicate to form a network. However, the very low Al/Na ratio indicates a high enrichment of Na relative to Al, which signifies high Na_2_O content (excess Na^+^ ions) and disrupts NASH formation.^68^ Furthermore, the high water/solid ratio of 0.49 weakens the microstructure adhesion with the presence of large pores and microcracks.^98^ At the identical SiO_2_/Al_2_O_3_ ratio of 3.5 with a relatively high Al_2_O_3_/Na_2_O ratio of 1.4 and a low water/solid ratio of 0.32 (mix #24), the SEM images reveal a significant decrease in the amount of unreacted metakaolin, simultaneously with the formation of a compact/dense matrix (Fig. 8c1 and 2). Table 6 shows a Si/Al ratio of 1.89 and the lowest Al/Na ratio of 0.97, representing the most promising elemental balance among the analyzed specimens. The relatively high Si/Al ratio indicates sufficient silicate polymerization, while the balanced Al/Na ratio implies the presence of an adequate amount of silica and Na^+^ ions that balance the charges of the geopolymeric products, promoting the formation of cross-linked phases, rather than remaining as excess sodium.^40,68^ Furthermore, Fig. 8c3 shows that mix #24 has the highest Si and Al wt%, confirming their effective incorporation in the formation of the geopolymeric network, agreeing with its superior compressive strength of 50.80 MPa.

In the case of mix #31, which has a high SiO_2_/Al_2_O_3_ ratio of 4.1, a low Al_2_O_3_/Na_2_O ratio of 0.85 and a water/solid ratio of 0.45, the SEM images (Fig. 8d1 and 2) show well-dispersed rounded geopolymeric products, as detected by Papa *et al.*^99^ The EDX analysis (Fig. 8d3 and Table 6) shows that Si/Al and Al/Na are 2.01 and 0.70, respectively. The relatively high Si and Na content confirms the silicate/sodium-rich matrix, precipitating heterogeneous products. Furthermore, the high water/solid ratio increases the porosity, resulting in a decrease in the compressive strength by 57.75% with respect to mix #24.^40,49,100^

#### 3.4.5. Texture characteristics

A texture characteristics study was conducted to analyse the pore structure of selected metakaolin geopolymers (mix #11, 19, 24 and 31) to describe the basic function of the different molar ratios (SiO_2_/Al_2_O_3_ and Al_2_O_3_/Na_2_O) and their microstructure and textural qualities. The main textural characteristics measured were specific surface area (SA, m^2^ g^−1^), total pore volume (*V*_t_, cm^3^ g^−1^), monolayer capacity (*V*_m_, cm^3^ g^−1^) and maximum pore diameter (dp_max_, nm). The term “dp_max_” generally refers to the most probable or representative pore size within a measured distribution.^63,65,80,101^ The mechanical performance of geopolymer matrices is significantly influenced by the size, distribution and connectivity of pores, as well as the total pore volume. Therefore, the dp_max_ is considered one of the key factors affecting mechanical performance.^89,102,103^

**Fig. 9:**
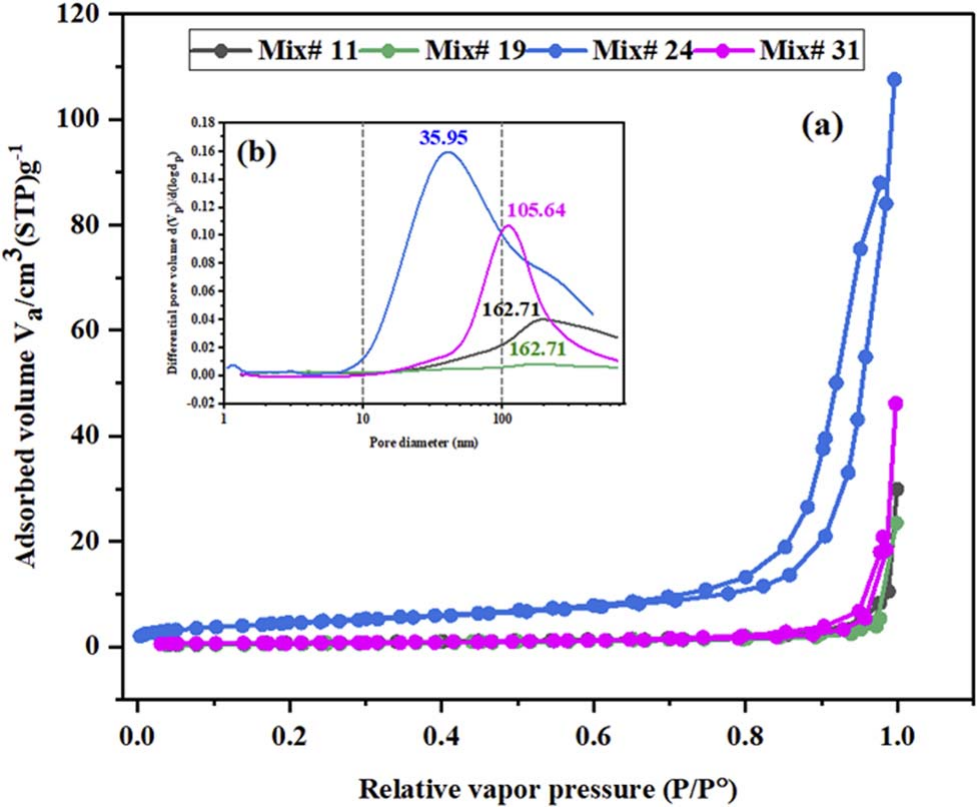
(a) N_2_-adsorption/desorption isotherms and (b) pore-size distribution curves of the geopolymer mix #11, 19, 24 and 31 after 28 days of hydration.

From Fig. 9a, b and Table 7, mix #24 exhibits the highest SA (16.31 m^2^ g^−1^), *V*_t_ (0.147 cm^3^ g^−1^) and *V*_m_ (3.75 cm^3^ g^−1^). According to the dp_max_ value of 35.59 nm, the IUPAC classification is a mesopore-like structure (the pore diameter lies between 2 and 50 nm). This indicates that the pore structure system of mix #24 mainly consists of fine gel-related mesoporosity rather than large capillary voids or macrodefects. This refined pore structure aligns with forming a compact and well-condensed sodium aluminosilicate geopolymer binding gel.^104^ Accordingly, the high surface area of mix #24 results from the fine pore structure of the geopolymer gel, and its small pore diameter suggests efficient microstructural densification. This is also considered good evidence for the role of balancing the SiO_2_/Al_2_O_3_ of 3.5 and Al_2_O_3_/Na_2_O of 1.40 and a low water/solid ratio of 0.32 in the formation of a continuous NASH gel in the form of a three-dimensional network structure with fine mesoporosity. This matches the superior hydraulic nature of mix #24, which was reflected in the greater mechanical performance of 50.8 MPa.^40,68^ On the other hand, mix #19, although it has the same SiO_2_/Al_2_O_3_ ratio of 3.5, the low Al_2_O_3_/Na_2_O ratio of 0.85 and high water/solid ratio of 0.49 have a significant detrimental impact on the compressive strength (3.04 MPa). The high Na^+^ ions, which disrupt the geopolymerization process, and the high water content, which increases the porosity, are the main reasons, as illustrated above.^68^ These discussions are supported by texture analysis, as in Table 7; this specimen has a low SA (2.17 m^2^ g^−1^), *V*_t_ (0.27 cm^3^ g^−1^) and *V*_m_ (0.50 cm^3^ g^−1^), as well as a high dp_max_ (162.71 nm). This suggests that the pore structure was dominated by macropores rather than by fine-pore gel, disrupting gel continuity and resulting in poor packing density, matching the SEM images. These large voids and microcracks act as stress concentrations and reduce the effective load-bearing capacity, explaining the very low compressive strength of mix #19 (3.04 MPa).

**Table 7:** Main textural parameters of the geopolymer specimens

Composite	SA (m^2^ g^−1^)	*V*_t_ (cm^3^ g^−1^)	*V*_m_ (cm^3^ g^−1^)	dp_max_ (nm)	Isotherm type
Mix #11	2.46	0.022	0.57	162.71	IV/H3
Mix #19	2.17	0.027	0.50	162.71	IV/H3
Mix #24	16.31	0.147	3.75	35.59	IV/H3
Mix #31	2.69	0.045	0.62	105.64	IV/H3

**Fig. 10:**
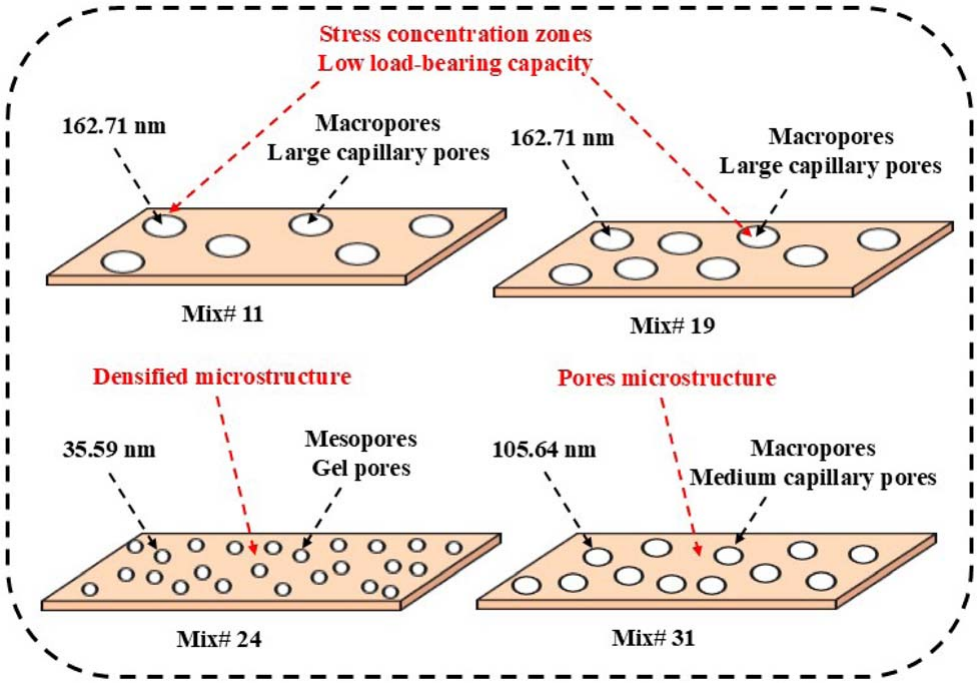
Schematic of the texture characteristics for selected specimens.

Mix #11 has approximately the same SA, *V*_t_, *V*_m_ and dp_max_ (2.46 m^2^ g^−1^, 0.022 cm^3^ g^−1^, 0.57 cm^3^ g^−1^, 162.71 nm, respectively) as mix #19. The dp_max_ denotes the formation of poorly developed gel networks consisting of macropores, in line with the formation of a heterogeneous matrix. Although the water/solid ratio of mix #11 was relatively low (0.32), the presence of a moderate amount of SiO_2_ (SiO_2_/Al_2_O_3_ ratio = 3.10) may not be sufficient to develop the formation of three-dimensional network phases, resulting in a moderate compressive strength of 12.56 MPa. Finally, for mix #31, Table 7 shows that it has SA, *V*_t_, *V*_m_ and dp_max_ of 2.69 m^2^ g^−1^, 0.045 cm^3^ g^−1^, 0.62 cm^3^ g^−1^, and 105.64 nm, respectively. This clarifies that the SA, *V*_t_ and *V*_m_ values of mix #31 are higher than those of mix #11 and 19 and lower than those of mix #24. Also, the dp_max_ is lower than those of mix #11 and 19 and higher than that of mix #24. These data match the compressive-strength value of mix #31 (21.49 MPa), which is moderate among the other mixes. In this case, increasing the SiO_2_ beyond the required value, which hinders the metakaolin geopolymerization, and a high water/solid ratio may be reasons behind its low strength compared to mix #24.^40,49^Fig. 10 shows a schematic representation that clarifies the impact of the mix-design parameters on the texture characteristics.

## 4. Conclusion

This study aims to optimize the SiO_2_/Al_2_O_3_ (2.74 to 4.1) and Al_2_O_3_/Na_2_O (0.75–1.50) molar ratios to obtain a metakaolin geopolymer with superior mechanical performance. The results were supported by different analysis techniques, such as XRD, TGA/DTG, SEM and BET/BJH models. The following conclusions can be drawn:

• Calcination of kaolin at 700 °C for 1 h is a sufficient condition to produce reactive metakaolin. The TGA analysis shows a near-complete dehydroxylation, reaching 96.20%. XRD and SEM confirm the transformation of kaolinite phases into amorphous metakaolin.

• The optimum Al_2_O_3_/Na_2_O ratio depends on the SiO_2_/Al_2_O_3_ ratio; increasing the SiO_2_/Al_2_O_3_ ratio requires a lower Al_2_O_3_/Na_2_O ratio to avoid the presence of Na^+^ ions that disturbs the geopolymerization reaction. The optimum Al_2_O_3_/Na_2_O ratio at SiO_2_/Al_2_O_3_ ratios of 3.5, 3.7, 3.9 and 4.1 are 1.4, 1.3, 1.0 and 0.85, respectively.

• The adequate SiO_2_/Al_2_O_3_ ratio indicates the presence of active silica in the geopolymeric matrix that serves as active sites for dissolution of aluminosilicate precursors. This is confirmed by the existence of low intensities of unreacted metakaolin phases in XRD patterns at a SiO_2_/Al_2_O_3_ ratio of 3.5 (mix #24).

• Optimizing SiO_2_/Al_2_O_3_ and Al_2_O_3_/Na_2_O ratios at 3.5 and 1.4 produces thermally stable three-dimensional phases, which is confirmed by the high mass-loss% of mix #24 in TGA analysis.

• The metakaolin geopolymer prepared with SiO_2_/Al_2_O_3_ and Al_2_O_3_/Na_2_O ratios of 3.5 and 1.4, respectively, yields a dense matrix with high-quality texture characteristics (highest surface area, total pore volume and monolayer capacity, as well as lowest maximum pore diameter).

• Metakaolin with superior mechanical performance can be achieved by co-optimising SiO_2_/Al_2_O_3_, Al_2_O_3_/Na_2_O, H_2_O/Na_2_O and water/solid ratios at 3.5, 1.4, 9.5 and 0.32, respectively.

## Author contributions

Hisham Abdeen: data curation, methodology, writing – original draft, writing – review and editing, validation, investigation. Alaa Mohsen: conceptualization, data curation, methodology, writing – original draft, writing – review and editing, validation, investigation, supervision. AbdelMonem Soltan: conceptualization, data curation, methodology, writing – original draft, writing – review and editing, validation, investigation, supervision. Mohamed Kohail: conceptualization, data curation, methodology, writing – original draft, writing – review and editing, validation, investigation, supervision.

## Conflicts of interest

There are no conflicts to declare.

## Data Availability

The data supporting the findings of this study are available from the corresponding author upon reasonable request.
